# New-Onset Age of Nonalcoholic Fatty Liver Disease and Cancer Risk

**DOI:** 10.1001/jamanetworkopen.2023.35511

**Published:** 2023-09-25

**Authors:** Chenan Liu, Tong Liu, Qingsong Zhang, Pingping Jia, Mengmeng Song, Qi Zhang, Guotian Ruan, Yizhong Ge, Shiqi Lin, Ziwen Wang, Hailun Xie, Jinyu Shi, Ruiqin Han, Yue Chen, Xin Zheng, Liuyi Shen, Li Deng, Shouling Wu, Hanping Shi

**Affiliations:** 1 Department of Gastrointestinal Surgery/Clinical Nutrition, Beijing Shijitan Hospital, Capital Medical University, Beijing, China; 2 National Clinical Research Center for Geriatric Diseases, Xuanwu Hospital, Capital Medical University, Beijing, China; 3 Key Laboratory of Cancer Food for Special Medical Purposes for State Market Regulation, Beijing, China; 4 Beijing International Science and Technology Cooperation Base for Cancer Metabolism and Nutrition, Beijing, China; 5 Department of General Surgery, Kailuan General Hospital, Tangshan, China; 6 Cardiovascular Research Institute, University of California, San Francisco; 7 Department of Genetics, Yale University School of Medicine, New Haven, Connecticut; 8 State Key Laboratory of Medical Molecular Biology, Department of Biochemistry and Molecular Biology, Institute of Basic Medical Sciences, Chinese Academy of Medical Sciences and Peking Union Medical College, Beijing, China; 9 Department of Pathology, Shanxi Medical University, Taiyuan, Shanxi, China; 10 Department of Cardiology, Kailuan General Hospital, Tangshan, China

## Abstract

**Importance:**

The onset age of nonalcoholic fatty liver disease (NAFLD) is decreasing, and whether earlier ages of NAFLD onset are associated with increased cancer risk is currently unclear.

**Objective:**

To explore the association between NAFLD new-onset age and cancer risk.

**Design, Setting, and Participants:**

This cohort study was conducted among 179 328 participants included in the Kailuan Cohort Study between 2006 and 2021. In total, 46 100 incident NAFLD cases were identified. For each case, a participant matched by age (older or younger by 1 year) and sex was randomly selected to create a new matched study cohort. Data were analyzed from December 2022 through April 2023.

**Exposure:**

Onset of NAFLD.

**Main Outcomes and Measures:**

The association between the onset age of NAFLD and the risk of different cancer types was evaluated using weighted Cox regression models. Population-attributable fractions (PAFs) were used to quantify the association of NAFLD with cancer risk at different ages.

**Results:**

Among 63 696 participants (mean [SD] age, 51.37 [12.43] years; ‭10 932 females [17.2%] and ‭52 764 males [82.8%]), 31 848 individuals had NAFLD and 31 848 individuals were in the control group. During a median (IQR) follow-up of 10.16 (7.89-11.67) years, 2415 patients were diagnosed with cancer. Compared with the matched group, patients aged less than 45 years at NAFLD onset exhibited a higher risk of cancer (average hazard ratio [AHR], 1.52; 95% CI, 1.09-2.12), and as the onset age of NAFLD increased, the cancer risk decreased (ages 45-54 years: AHR, 1.50; 95% CI, 1.15-1.97; ages 55-64 years: AHR, 1.13; 95% CI, 0.97-1.33; ages >65 years: AHR, 0.75; 95% CI, 0.45-1.27; *P* for interaction < .001). Among patients aged less than 45 years at NAFLD onset, cancers were mainly digestive system and lung cancers, with AHR values of 2.00 (95% CI, 1.08-3.47) and 2.14 (95% CI, 1.05-4.36), respectively. PAFs also showed that in patients aged less than 45 years at NAFLD onset, 17.83% (95% CI, 4.92%-29.86%) of cancer risk was attributable to NAFLD.‬‬‬‬

**Conclusions and Relevance:**

This study found that NAFLD was associated with increased cancer risk and there was an interaction with onset age, such that the younger the onset age of NAFLD, the greater the cancer risk.

## Key Points

**Question:**

Is the earlier onset age of nonalcoholic fatty liver disease (NAFLD) associated with increased risk of cancer?

**Findings:**

In this cohort study of 63 696 participants with different ages, NAFLD was associated with increased cancer risk and younger onset age of NAFLD was associated with greater risk of cancer.

**Meaning:**

This study found that early-onset NAFLD was associated with increased risk of cancer, suggesting that timely intervention in the progression of NAFLD may be associated with decreased incidence of NAFLD-related cancers and reduced burden on public health.

## Teaser

This cohort study investigates the association between early onset of nonalcoholic fatty liver disease and risk of cancer.

## Introduction

Nonalcoholic fatty liver disease (NAFLD), associated with significant liver damage, occurs in approximately 25% of the global population. Estimated adult NAFLD prevalence is projected to reach 33.5% by 2030, posing a serious global public health threat.^1,2^ With strong association with type 2 diabetes and metabolic syndromes, NAFLD incidence increases alongside diabetes and obesity prevalence.^3,4^ The harm caused by NAFLD also deepens gradually with its progression. NAFLD and its complications, including nonalcoholic steatohepatitis (NASH) and cirrhosis, impair liver function and are associated with other systemic diseases, such as cardiovascular disease and chronic kidney disease.^5,6^ Several large cohort studies also found that NAFLD was associated with cancer risk. Mantovani and Karl et al^6,7^ found that patients with NAFLD had a 1.2- to 15-fold higher risk of liver cancer, gastrointestinal cancers, and all cancer types. NAFLD-induced NASH has emerged as a leading cause of liver cancer deaths, surpassing hepatitis B and C virus.^8^ Therefore, the sustained increase in NAFLD incidence has resulted in new concerns.

Importantly, the population with NAFLD is becoming younger. Among patients with chronic liver disease–related deaths, one-third experienced NAFLD at younger than age 30 years.^9^ These outcomes suggest that attention must be paid to the association of new-onset NAFLD at different ages with the risk of developing other diseases, such as cancer. However, most studies have focused on health outcomes associated with the prevalent NAFLD age rather than NAFLD new-onset ages. We hypothesized that the younger onset age of NAFLD would be associated with higher cancer risk. Therefore, this study explored the association between the age of new-onset NAFLD and the risk of all cancer types in a large prospective cohort.

## Methods

This cohort study was approved by the ethics committee of Kailuan General Hospital and Beijing Shijitan Hospital, and the study followed the guidelines of the Helsinki Declaration. All participants voluntarily participated in this study after receiving a detailed introduction to the research design and provided written informed consent by themselves or through their legal representatives. This study is reported following the Strengthening the Reporting of Observational Studies in Epidemiology (STROBE) reporting guideline.

### Study Design and Participants

Study participants were selected from the Kailuan Cohort Study, an ongoing prospective cohort study that began in June 2006 (eMethods in Supplement 1).^10^ For this study, individuals who had undergone physical examinations at least twice between 2006 and 2017 and completed follow-up examinations were initially selected. A total of 179 328 participants were included. Those with a preexisting diagnosis or history of NAFLD, cancer, heavy alcohol consumption (alcohol intake ≥30 g/d for males and ≥20 g/d for females), hepatitis B virus infection, or other liver diseases were excluded. The final case group consisted of 46 100 patients who developed new-onset NAFLD between 2006 and 2017. After excluding patients with missing covariate data at the time of diagnosis, 35 860 patients remained.

**Figure 1.**
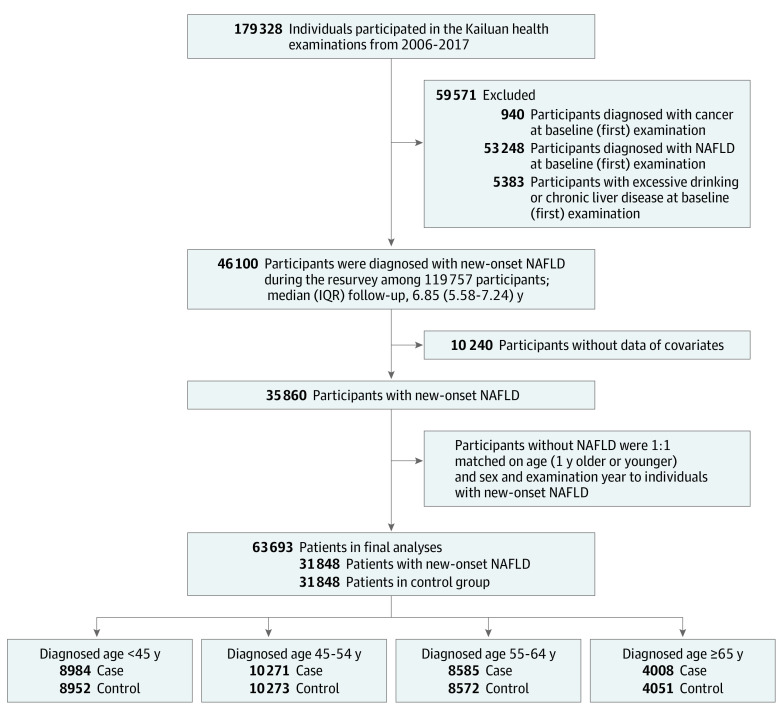
Study Flowchart NAFLD indicates nonalcoholic fatty liver disease.

The matched group was formed by randomly selecting healthy individuals who had participated in the physical examination in the same year as the case was diagnosed and were matched 1:1 based on age (older or younger by 1 year) and sex of patients in the case group. The follow-up of this event case started when new-onset NAFLD was identified. For example, we identified a male patient in 2010 with new-onset NAFLD at age 45 years. At the same time, matched individuals were randomly selected from the group of individuals without NAFLD who had participated in the physical examination in 2010 at ages 44 to 46 years; both groups were followed up starting in 2010. After a median (IQR) follow-up of 6.85 (5.58-7.24) years, 31 848 pairs of participants were included in the study (Figure 1) and were grouped according to age.

### Definition of New-Onset NAFLD

NAFLD was defined as (1) the presence of hepatic steatosis diagnosed through imaging (eMethods in Supplement 1), (2) no history of excessive alcohol consumption (ethanol intake <140 g/wk for males and <70 g/wk for females) in the past 12 months, and (3) no competing etiologies for hepatic steatosis and no coexisting causes for chronic liver disease.^11^ Moreover, we had to ensure that participant NAFLD was new onset. Therefore, participants had to have attended at least 2 physical examinations between 2006 and 2017, with the first examination showing no NAFLD and NAFLD appearing in the subsequent examination (for example, no NAFLD detected in a participant who underwent a physical examination for the first time in 2008 but NAFLD diagnosed in a 2010 examination). New-onset NAFLD was diagnosed on the basis of 57 conditions (eTable 1 in Supplement 1). A sensitivity analysis was conducted on metabolic dysfunction–associated fatty liver disease (MAFLD) based on the international expert consensus and guidelines of the Asia-Pacific Association for the Study of the Liver (eMethods in Supplement 1).

### Outcome and Covariates

Cancer occurrence was considered the study outcome (eMethods in Supplement 1). The cancer diagnosis was determined by at least 2 well-trained doctors through pathology or imaging and recorded by the *International Statistical Classification of Diseases and Related Health Problems, Tenth Revision* (*ICD-10*) (eMethods in Supplement 1). The follow-up period was defined as the time from the date of diagnosis of NAFLD to the occurrence of cancer or death or the date of the last follow-up (December 31, 2021), whichever came first. Covariates included demographic data, questionnaire responses, laboratory indicators, and anthropometry (eMethods in Supplement 1).

### Statistical Analysis

Continuous variables were summarized as mean (SD) for normally distributed data and compared using *t* tests or analysis of variance. Skewed continuous variables were presented as median (IQR) and compared using the Kruskal-Wallis test. Categorical variables were reported as percentages and compared using the χ^2^ test. Cox regression was used to analyze the risk of cancer incidence. Because our model did not meet the assumption of proportional hazard ratios, we used a weighted Cox regression model to calculate the average hazard ratio (AHR) and 95% CI for cancer incidence. To further assess the association of NAFLD exposure with cancer incidence across age groups, population attributable fractions (PAFs) were calculated^12,13^ (eMethods in Supplement 1).

Subgroup analyses were based on inflammation and liver function (eMethods in Supplement 1). Sensitivity analyses were performed to assess the robustness of results. Participants who were diagnosed with cancer in the first year of follow-up were excluded to avoid causation inversion, although this is a prospective study. Propensity score matching was performed to eliminate the potential association of other factors with outcomes. Finally, we excluded participants who used medications and performed regular physical activity. During the follow-up period, death may act a competing event. Competing risk models (Fine and Gray model) were applied for reducing this bias, including the cause-specific hazards function and subdistribution hazards function. Statistical analyses were performed using SAS statistical software version 9.4 (SAS Institute) and R statistical software version 4.2.0 (R Project for Statistical Computing). A 2-sided *P* < .05 was considered statistically significant. Data were analyzed from December 2022 through April 2023.

## Results

### Baseline Characteristics

Among 63 696 participants (mean [SD] age, 51.37 [12.43] years; ‭10 932 females [17.2%] and ‭52 764 males [82.8%]), the sex and age of matched (31 848 individuals) and case (31 848 individuals) groups were matched (Table). In the case group, there were 8984 patients aged less then 45 years, 10 271 patients aged 45 to 54 years, 8585 patients aged 55 to 64 years, and 4008 patients aged 65 years or older. Compared with the matched group, patients with new-onset NAFLD often lacked regular physical activity; had a higher prevalence of hypertension; more frequently had overweight and obesity; had higher mean (SD) waist circumference and levels of triglyceride, total cholesterol, hypersensitive C-reactive protein, and alanine aminotransferase (ALT) and lower mean (SD) levels of high-density lipoprotein cholesterol and total bilirubin. With an increase in NAFLD new-onset age, the proportions of hypertension and diabetes were higher and the mean (SD) body mass index (calculated as weight in kilograms divided by height in meters squared) were reduced. Moreover, mean (SD) waist circumference and levels of total cholesterol, hypersensitive C-reactive protein, and total bilirubin increased while levels of triglyceride and ALT decreased as the NAFLD new-onset age increased. In addition, we compared baseline characteristics between excluded participants and those involved in the study (eTable 2 in Supplement 1). There were no significant differences between groups in factors such as age, sex, smoking, hypertension, and diabetes.‬‬‬‬

**Table.** Baseline Clinical Characteristics of Participants

Characteristic^a^	Total patients, No. (%) (N = 63 696)			*P* value	Patients by NAFLD onset age, No. (%)				*P* value
	Control group (n = 31 848)		New-onset NAFLD (n = 31 848)		<45 y (n = 8984)	45-54 y (n = 10 271)	55-64 y (n = 8585)	≥65 y (n = 4008)	
Age, y, mean (SD)	51.37 (12.43)	51.37 (12.43)		.96	36.04 (6.57)	50.32 (2.28)	59.30 (2.76)	71.42 (5.16)	<.001
Sex									
Female	5466 (17.2)	5466 (17.2)		>.99	1242 (13.8)	2211 (21.5)	1498 (17.4)	515 (12.8)	<.001
Male	26 382 (82.8)	26 382 (82.8)			7742 (86.2)	8060 (78.5)	7087 (82.6)	3493 (87.2)	
Regular physical activity	5176 (16.3)	4874 (15.3)		.001	1087 (12.1)	1261 (12.3)	1668 (19.4)	858 (21.4)	<.001
Current smoker	6864 (21.6)	6787 (21.3)		.46	2406 (26.8)	1800 (17.5)	1681 (19.6)	899 (22.4)	<.001
GSD	672 (2.1)	726 (2.3)		.15	85 (0.9)	204 (2.0)	265 (3.1)	172 (4.3)	<.001
Gallbladder polyps	560 (1.8)	548 (1.7)		.74	160 (1.8)	176 (1.7)	154 (1.8)	58 (1.4)	.52
Diabetes	3164 (9.9)	3152 (9.9)		.88	363 (4.0)	1086 (10.6)	1116 (13.0)	587 (14.6)	<.001
Hypertension	12 442 (39.1)	14 518 (45.6)		<.001	2434 (27.1)	4657 (45.3)	4875 (56.8)	2552 (63.7)	<.001
BMI									
<24.00	13 545 (42.5)	8511 (26.7)		<.001	2074 (23.1)	2843 (27.7)	2348 (27.4)	1246 (31.1)	<.001
24.00-27.99	13 036 (40.9)	16 867 (53.0)			4835 (53.8)	5479 (53.3)	4549 (53.0)	2004 (50.0)	
≥28.00	5267 (16.5)	6470 (20.3)			2075 (23.1)	1949 (19.0)	1688 (19.7)	758 (18.9)	
Mean (SD)	24.84 (3.44)	25.78 (2.97)		<.001	26.06 (3.02)	25.67 (2.97)	25.75 (2.92)	25.53 (2.99)	<.001
Waist circumference, mean (SD), cm	86.88 (9.87)	89.22 (9.11)		<.001	88.86 (8.47)	88.99 (8.84)	89.62 (9.68)	89.74 (9.82)	<.001
Triglyceride, median (IQR), mg/dL	109.86 (77.97-161.25)	129.36 (90.37-194.03)		<.001	139.10 (96.57-215.30)	136.44 (93.12-208.21)	122.27 (87.71-177.20)	112.52 (80.63-158.59)	<.001
Total cholesterol, median (IQR, mg/dL)	189.05 (165.85-213.40)	196.39 (173.58-222.30)		<.001	189.82 (167.40-213.02)	197.94 (175.52-196.39)	200.65 (156.96-227.71)	196.78 (173.20-223.07)	<.001
HDL-c, median (IQR), mg/dL	53.35 (45.42-63.40)	52.58 (44.07-63.02)		<.001	51.42 (43.30-61.08)	54.12 (45.23-64.95)	51.80 (44.07-63.02)	52.19 (44.07-62.24)	<.001
hs-CRP, median (IQR), mg/dL	0.11 (0.05-0.25)	0.14 (0.07-0.29)		<.001	0.13 (0.06-0.26)	0.14 (0.00-0.28)	0.14 (0.07-0.3)	0.16 (0.07-0.36)	<.001
Total bilirubin, median (IQR), mg/dL	0.77 (0.60-0.97)	0.76 (0.60-0.96)		<.001	0.73 (0.57-0.93)	0.75 (0.59-0.95)	0.79 (0.62-0.99)	0.81 (0.63-1.01)	<.001
ALT, median (IQR), U/L	19.0 (14.0-26.0)	20.0 (15.0-28.0)		<.001	23.00 (16.0-34.0)	20.00 (15.0-28.0)	19.00 (14.4-25.2)	17.00 (13.0-22.3)	<.001

Abbreviations: ALT, alanine aminotransferase; BMI, body mass index (calculated as weight in kilograms divided by height in meters squared); GSD, gallstone disease; HDL-c, high-density lipoprotein cholesterol; hs-CRP, hypersensitive C-reactive protein; NAFLD, nonalcoholic fatty liver disease.

SI conversion factors: To convert alanine aminotransferase to microkatals per liter, multiply by 0.0167; HDL-c and total cholesterol and to millimoles per liter, multiply by 0.0259; hs-CRP to milligrams per liter, multiply by 10; total bilirubin to micromoles per liter, multiply by 17.104; triglyceride to millimoles per liter, multiply by 0.0113.

^a^ Characteristics were assessed in the examination cycle when new-onset NAFLD was first diagnosed.

During a median (IQR) follow-up of 10.16 (7.89-11.67) years, 2415 patients were diagnosed with cancer. By age group, the median (IQR) follow-up was 10.10 (7.02-11.03) years for participants aged less than 45 years, 10.11 (8.63-11.66) years for participants aged 45 to 54 years, 10.13 (7.84-12.25) years for participants aged 55 to 64 years, and in 10.08 (7.81-11.60) years for participants aged 65 years or older at NAFLD onset. A total of 1134 and 1281 participants in the matched and case groups, respectively, developed cancers (eTable 3 in Supplement 1).

### New-Onset NAFLD and Cancer Risk Across Age Groups

**Figure 2.**
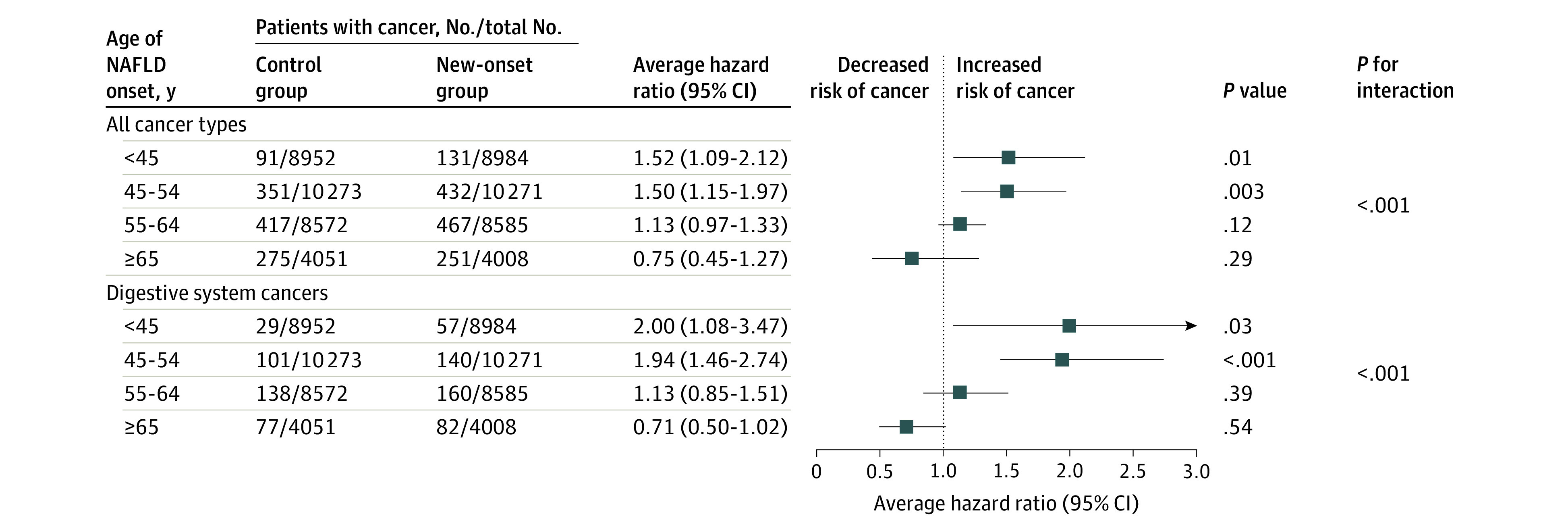
Risk of Cancer by Age Group Average hazard ratios with 95% CIs of all cancer types and digestive system cancers are presented among participants with new-onset NAFLD vs controls across age groups. The model was adjusted for age (continuous), sex (categorical), body mass index (continuous; calculated as weight in kilograms divided by height in meters squared), waist circumference (continuous), total cholesterol level (continuous), triglyceride level (continuous), total bilirubin level (continuous), hypersensitive C-reactive protein level (continuous), alanine aminotransferase level (continuous), smoking status (categorical), physical activity (categorical), hypertension (categorical), diabetes (categorical), gallbladder polyps (categorical), and gallstone disease (categorical). NAFLD indicates nonalcoholic fatty liver disease.

As shown in Figure 2, after adjustment for covariates, the risk of cancer incidence in patients aged younger than 65 years with new-onset NAFLD was higher than that in matched participants, including patients aged 55 to 64 years (AHR, 1.13; 95% CI, 0.97-1.33), with especially high AHRs in patients aged less than 45 years (AHR, 1.52; 95% CI, 1.09-2.12) or 45 to 54 years (AHR, 1.50; 95% CI, 1.15-1.97) at onset of NAFLD. Considering the association between NAFLD and digestive system cancers found in previous studies,^14^ we further calculated AHRs of digestive system cancers. Results were similar to those for all cancer types; the risk of digestive system cancers was higher in patients aged less than 45 years (AHR, 2.00; 95% CI, 1.08-3.47) and 45 to 54 years (AHR, 1.94; 95% CI, 1.46-2.74) at new-onset NAFLD. The risk of cancer incidence decreased with an increase in the onset age of NAFLD; when the age of new-onset of NAFLD was 65 years or older, compared with the matched group, the risk of all cancer types (AHR, 0.75; 95% CI, 0.45-1.27; *P* for interaction < .001) and digestive system cancers (AHR, 0.71; 95% CI, 0.50-1.02; *P* for interaction < .001) in the case group lost statistical significance.

We further clarified the association between new-onset NAFLD at different ages and the risk of different cancer types (eFigure 1 in Supplement 1). A similar trend was observed for liver cancer, colorectal cancer (CRC), and lung cancer. Younger age of NAFLD onset was associated with greater risk of liver and lung cancers. Among patients aged less than 45 years at new-onset NAFLD, the AHR of liver and lung cancers was 2.66 (95% CI, 1.16-6.11) and 2.14 (95% CI, 1.05-4.36), respectively. No significant differences were observed in the risk of CRC among patients who were younger at NAFLD onset (ages <45 years: AHR, 2.05; 95% CI, 0.68-8.87; ages 45-54 years: AHR, 2.89; 95% CI, 1.43-5.51). Similarly in urinary system cancers and gallbladder and extrahepatic bile duct cancers, no statistical differences were observed in AHRs, which could be limited by the sample size. We conducted subgroup analyses investigating the association between new-onset NAFLD and the risk of all cancer types, digestive system cancers, liver cancer, CRC, and lung cancer. At different inflammation levels, the risk of new-onset NAFLD and the aforementioned cancers continued to exhibit a trend of changing with age, and in patients with high inflammation levels, interactions with age were more pronounced (eTable 4 in Supplement 1). Among participants with higher ALT levels, the cancer risk decreased with an increase in the new-onset age of NAFLD, and these associations were modified by age (eTable 5 in Supplement 1).

### Additional Analyses

Results of a competing risk model exhibited that in subdistribution and cause-specific models, as the age of new-onset NAFLD decreased, the cancer risk increased. In all cancer types, digestive system cancers, liver cancer, CRC, and lung cancer, results of the competing risk analysis were more robust compared with main results (eTable 6 in Supplement 1).

In addition, we conducted several sensitivity analyses, excluding 63 176 patients who were diagnosed with cancers within the first year (eTable 7 in Supplement 1), 56 651 patients who performed regular physical activity, and 52 536 patients who took lipid-lowering drugs during the follow-up period (eTable 8 in Supplement 1) and using the propensity score–matching analysis among 53 778 patients (eTable 9 in Supplement 1). Results were similar to those of the primary analysis. Finally, comparing the association between the age of new-onset MAFLD and cancer risk, we found that it was similar to the primary finding (eTable 10 in Supplement 1).

### Population Attribution Fractions

Consistent with results of a weighted Cox regression analysis, PAFs of new-onset NAFLD decreased with age, which was observed for all cancer types, digestive system cancers, and 3 specific site cancers (liver cancer, CRC, and lung cancer) (eFigure 2 in Supplement 1). For example, the PAF of all cancer types began to decrease from 17.83% (95% CI, 4.92%-29.86%) among patients aged less than 45 years at new-onset NAFLD, which meant that if participants avoided NAFLD before age 45 years, their subsequent cancer risk may have decreased by 17.83%.

## Discussion

Our prospective cohort study of 63 696 participants found that patients with NAFLD onset before age 45 years had the highest risk of developing cancers, particularly digestive system cancers (liver cancer and CRC) and lung cancer. Notably, the risk of cancer decreased as the age at NAFLD onset increased. This suggests that preventing and reducing NAFLD in early life may be associated with significantly lower cancer prevalence.

Numerous studies have examined the association between NAFLD and the incidence of cancers and all-cause mortality. In a large retrospective study, Kanwal et al^15^ found that patients with NAFLD had a 7.62 times higher incidence of liver cancer compared with the sex-matched general population. The risk of other digestive system tumors has also been found to increase with NAFLD. Studies in 2020^16^ and 2019^17^ reported associations between NAFLD and the occurrence of colonic adenomatous polyps, CRC, and even CRC’s metastasis and poor prognosis. Furthermore, reports suggested an association between NAFLD and non–digestive system cancers. A meta-analysis^18^ involving 182 202 individuals found that NAFLD was associated with an increase in the risk of lung cancer by an additional 30%, even after adjusting for metabolic-related factors. Mechanically, as NAFLD progresses, lipid accumulation in liver cells leads to oxidative damage and DNA mismatch repair, which act as driving factors for cancer.^19^ The association between NAFLD and cancer is extremely close in mechanism and epidemiology.

However, few studies have investigated the interaction of age with the NAFLD-outcomes association. Contrary to traditional beliefs, the incidence of NAFLD is high even among children and young people. Compared with the matched group, young adults with NAFLD had significantly increased overall mortality and cancer-specific mortality rates, which were 5.88- and 15.60-fold higher, respectively, than in healthy young adults.^20^ Findings from a retrospective study^21^ suggested that the leading causes of death and liver-related mortality in patients with NAFLD changed with increasing age, and the incidence of liver-related deaths was higher in males younger than age 70 years. Another study^22^ described different roles of the same driver genes in different NAFLD onset ages, which suggested that age may play a crucial role in NAFLD. However, these studies mainly focused on patients with the prevalent NAFLD age. In contrast, our study focused on new-onset NAFLD during follow-up, which may have reduced potential prevalence-incidence bias.^23^

Our research highlights the dangers associated with early exposure to NAFLD. Patients with NAFLD in their early stage of life may experience metabolic disorders with sustained liver damage. In our sensitivity analysis, patients who had received treatment after diagnosis were excluded. Results revealed that in these patients who were exposed to NAFLD at an early stage, the subsequent risk of cancer was higher if they did not receive intervention and treatment. On further exploration, we found that risk factors, incidence, and characteristics of patients with NAFLD differed by age group. In Chinese populations, the highest incidence of NAFLD was in individuals aged younger than 50 years.^24^ Hence, this finding may hold importance in raising awareness and altering perceptions among the Chinese population regarding early and new-onset NAFLD. Similar to our findings, another study^25^ found that with an increase in the age of new-onset NAFLD, the risk of diabetes decreased, with the relative risk decreasing from 3.992 at age 30 years to 1.908 at age 60 years. The underlying mechanism may be related to long-term liver damage, fat accumulation, and decreased insulin sensitivity, which can also lead to the blocking of glucose metabolism pathways.^26^ Moreover, studies have reported an association between age and NAFLD from a mechanistic perspective. Chronic liver inflammation can lead to aging of liver cells. Cellular aging can lead to liver fat accumulation and hepatic steatosis, which further jointly promote liver fibrosis and hepatocellular carcinoma.^27,28^ These studies also reported that early and sustained exposure to NAFLD can impair liver function and metabolism, with associated systemic lesions.

Interestingly, our subgroup analysis revealed that in patients with high ALT levels, new-onset NAFLD was associated with all cancer types, digestive system cancers (liver cancer and CRC), and lung cancer, and a strong interaction was observed with age. This result is consistent with that of Natarajan et al.^29,30^ An earlier onset age of NAFLD was associated with a greater likelihood of increased ALT levels. Compared with patients with increased ALT levels, patients with NAFLD and sustained normal ALT levels exhibited a lower risk of liver cirrhosis and liver cancer.

MAFLD, the recently proposed term, is closely associated with metabolic disorders and has been associated with various cancers.^31^ Our study also found an association between MAFLD and cancers and for the first time, to our knowledge, described an association between age of MAFLD onset and cancer risk.

In the field of public health, Zhou et al^32^ found a concerning trend of younger NAFLD onset, with incidence among individuals younger than age 45 years surpassing that of older age groups in China over the last decade. We found that patients with early-onset NAFLD had a higher cancer risk, as confirmed by PAFs. These findings suggest that early screening and prevention of NAFLD may be crucial to reduce subsequent cancer occurrence. Increased awareness and urgent action are needed to control the NAFLD epidemic in China.

### Limitations

This study has several limitations. First, the Kailuan cohort consists primarily of male workers, leading to potential sex bias. Additionally, NAFLD diagnosis relied on ultrasound instead of liver biopsy, potentially missing mild NAFLD cases. The study also lacked data on liver fibrosis elastography measurement and blood biomarkers, such as fibrosis-4, for diagnosing and staging the degree of liver fibrosis. Moreover, some cancers had low incidence rates, showing no statistically significant difference by age group.

## Conclusions

This cohort study found an association between NAFLD and increased cancer risk, particularly in patients with early-onset NAFLD. The increasing incidence of NAFLD among younger populations highlights the underestimation of harmful outcomes associated with this condition. Our findings suggest that early control and intervention against NAFLD progression may be crucial to reduce the occurrence of NAFLD-related cancers and lessen the burden on public health.

## Notes

Supplement 1.
eMethods.
**eTable 1.** Determination of New-Onset Nonalcoholic Fatty Liver Disease **eTable 2.** Comparison of Baseline Characteristics Between Included and Excluded Participants **eTable 3.** Number of Onset Cases in 4 Groups **eTable 4.** Risk of Cancers by Hypersensitive C-Reactive Protein Level **eTable 5.** Risk of Cancers by Alanine Aminotransferase Level **eTable 6.** Association of New-Onset Nonalcoholic Fatty Liver Disease With Risk of Cancers in Competing Risk Analysis **eTable 7.** Risk of Overall Cancers Excluding 63 176 Participants With <1 y Follow-Up **eTable 8.** Risk of Overall Cancers Excluding 56 651 Participants With Regular Physical Activity, 52 536 Participants Regularly Taking Lipid-Lowering Drugs, and 62 595 Participants With Late Nonalcoholic Fatty Liver Disease Diagnosis **eTable 9.** Risk of Overall Cancers Among 53 778 Patients After Propensity Score Matching **eTable 10.** Risk of Overall Cancers Among 61 712 Participants With New-Onset Metabolic Dysfunction–Associated Fatty Liver Disease **eFigure 1.** Risk of Specific Cancers **eFigure 2. **Populations Attributable Fractions of Cancer Associated With New-Onset Nonalcoholic Fatty Liver Disease

Supplement 2.
Data Sharing Statement
