# Supplementary material for: New-Onset Age of Nonalcoholic Fatty Liver Disease and Cancer Risk
Source: JAMA Netw Open. 2023 Sep 25;6(9):e2335511. doi: 10.1001/jamanetworkopen.2023.35511 (PMC10520743; doi:10.1001/jamanetworkopen.2023.35511)
Supplement: Supplement 1. — eMethods. eTable 1. Determination of New-Onset Nonalcoholic Fatty Liver Disease eTable 2. Comparison of Baseline Characteristics Between Included and Excluded Participants eTable 3. Number of Onset Cases in 4 Groups eTable 4. Risk of Cancers by Hypersensitive C-Reactive Protein Level eTable 5. Risk of Cancers by Alanine Aminotransferase Level eTable 6. Association of New-Onset Nonalcoholic Fatty Liver Disease With Risk of Cancers in Competing Risk Analysis eTable 7. Risk of Overall Cancers Excluding 63 176 Participants With <1 y Follow-Up eTable 8. Risk of Overall Cancers Excluding 56 651 Participants With Regular Physical Activity, 52 536 Participants Regularly Taking Lipid-Lowering Drugs, and 62 595 Participants With Late Nonalcoholic Fatty Liver Disease Diagnosis eTable 9. Risk of Overall Cancers Among 53 778 Patients After Propensity Score Matching eTable 10. Risk of Overall Cancers Among 61 712 Participants With New-Onset Metabolic Dysfunction–Associated Fatty Liver Disease eFigure 1. Risk of Specific Cancers eFigure 2. Populations Attributable Fractions of Cancer Associated With New-Onset Nonalcoholic Fatty Liver Disease [file jamanetwopen-e2335511-s001.pdf]

Supplemental Online Content

Liu C, Liu T, Zhang Q, et al. New-onset age of nonalcoholic fatty liver disease and cancer risk. *JAMA Netw Open*. 2023;6(9):e2335511. doi:10.1001/jamanetworkopen.2023.35511

eMethods.

eTable 1. Determination of New-Onset Nonalcoholic Fatty Liver Disease

eTable 2. Comparison of Baseline Characteristics Between Included and Excluded Participants

eTable 3. Number of Onset Cases in 4 Groups

eTable 4. Risk of Cancers by Hypersensitive C-Reactive Protein Level

eTable 5. Risk of Cancers by Alanine Aminotransferase Level

eTable 6. Association of New-Onset Nonalcoholic Fatty Liver Disease With Risk of Cancers in Competing Risk Analysis

eTable 7. Risk of Overall Cancers Excluding 63 176 Participants With <1 y Follow-Up

eTable 8. Risk of Overall Cancers Excluding 56 651 Participants With Regular Physical Activity, 52 536 Participants Regularly Taking Lipid-Lowering Drugs, and 62 595 Participants With Late Nonalcoholic Fatty Liver Disease Diagnosis

eTable 9. Risk of Overall Cancers Among 53 778 Patients After Propensity Score Matching

eTable 10. Risk of Overall Cancers Among 61 712 Participants With New-Onset Metabolic Dysfunction–Associated Fatty Liver Disease

eFigure 1. Risk of Specific Cancers

eFigure 2. Populations Attributable Fractions of Cancer Associated With New-Onset Nonalcoholic Fatty Liver Disease

This supplemental material has been provided by the authors to give readers additional information about their work.

© 2023 Liu C et al. *JAMA Network Open.*

eMethods

1.This study was initiated by the Kailuan Group, a large enterprise involved in various fields such as coal, healthcare, finance, and manufacturing. Since 2006, employees of this enterprise have been continuously invited to undergo physical examinations at the Kailuan General Hospital and its 11 affiliated hospitals. We collected their physical examination data, questionnaire interview data, dietary information, laboratory test results, etc. biennially, and conducted annual follow-ups. Trained doctors or nurses conducted all surveys, and all participants signed informed consent. Due to the fact that this study is based on the largest tertiary hospital in Tangshan City, our database also connects provincial vital statistics data, Tangshan Medical Insurance System, and Kailuan Social Security Information System.

2. The diagnosis of NAFLD is based on B-ultrasound. In imaging diagnosis of China, patients' ultrasonography need to meet at least two of the following three conditions: (i) increased echogenicity of the liver near field region with deep attenuation of the ultrasound signal; (ii) hyperechogenity of liver tissue ("bright liver"), as often compared to hypoechogenity of the kidney cortex; and (iii) vascular blurring [1,2].

The severity of steatosis was differentiated by ultrasonography: mild (diffuse increase in fine echoes in liver parenchyma), moderate (diffuse increase in fine echoes with impaired visualization of the intrahepatic vessel borders and diaphragm), and severe (diffuse increase in fine echoes with nonvisualization of the intrahepatic vessel borders and diaphragm)[3].

Abdominal ultrasonography was routinely performed by experienced radiologists using a high-resolution B-mode topographical ultrasound system with a 3.5 MHz probe (ACUSON X300, Siemens, Germany) in the Kailuan study.

3. The diagnosis of MAFLD first needs to confirm liver steatosis based on imaging diagnosis, and then it is accompanied by any of the following three conditions, overweight/obesity, diabetes or the risk of metabolic abnormalities [4,5]. The metabolic risk abnormalities were defined as the presence of two or more of the following conditions: (1) Waist circumference ⩾90 in men and 80 cm in women. (2) Blood pressure ⩾130/85 mmHg or specific drug treatment. (3) TG ⩾1.70 mmol/l or specific drug treatment. (4) HDL-C <1.0 mmol/L for men and <1.3 mmol/L for women. (5) Prediabetes (i.e., fasting glucose levels 5.6 to 6.9 mmol/L, or 2-hour post-load glucose levels 7.8 to 11.0 mmol/L or HbA1c 5.7% to 6.4%. (6) Homeostasis model assessment-insulin resistance score ⩾2.5. (7) C-reactive protein (CRP) level >2 mg/L.

4. The Kailuan cohort was followed up by using face-to-face questionnaires and electronic medical record systems and reviewing death certificates from the Provincial Vital Statistics Offices, Tangshan medical insurance system, and Kailuan Social Security Information System [6].

5. The cancer types included were as follows: digestive system cancers [esophageal cancer (C15), gastric cancer (C16), small intestine cancer (C17), colorectal cancer (CRC; C18–C21), liver cancer (C22.0), pancreatic cancer (C25), gallbladder and extrahepatic bile duct cancer (C23–C24)], lung cancer (C34), urinary system tumors [kidney cancer (C64–C65), bladder cancer (C67)], lymphoma (C81–C89) and leukemia (C90–C96), gender-specific tumors [breast cancer (C50), cervix cancer (C53), uterus cancer (C54–C55), ovarian cancer (C56), prostate cancer (C61)], and other cancers.

6.Among them, regular physical activity was defined as ≥30 min/time, ≥ 3 times/week. A current smoker was defined as one who smokes at least one cigarette a day and has been smoking for at least 6 months. Sedentary was categorized based on self-reported time as <4 hours, 4-8 hours, and >8 hours. High-fat diet refers to a dietary pattern characterized by a higher intake of fatty acids but low in fiber, vitamins, and minerals, such as excessive consumption of fried foods, high-fat dairy products, pastries, and desserts. Salt consumption was self-reported and classified into three categories: low (<6 g/day), medium (6-9 g/day) or high (≥10 g/day). All laboratory indicators were measured after analysis with the automatic analyzer (Hitachi 747; Hitachi, Tokyo, Japan) in the central laboratory of Kailuan General Hospital.

7.PAFs indicate the proportion of disease-induced morbidity (or death) owing to certain exposure in the total morbidity (or death) of the population. Calculation method of PAF: In this study, we use the Miettinen formula: PAF=pc (1 − 1/RR). RR represents the relative risk, and pc represents the prevalence of case exposure. This calculation method is well-suited for cohort studies and can compute PAFs that account for multiple exposure levels or confounding factors [7]. "AF" package (R 4.2.0) is used to calculate PAF.

© 2023 Liu C et al. *JAMA Network Open.*

8.The participants were divided into the high inflammation group (hs-CRP ≥ 3 mg/L) and the low inflammation group (hs-CRP < 3 mg/L). Because of the limited sample size, the number of patients with abnormal liver function (>40 U/L) was too small, and we, therefore, divided the participants into the high ALT group (≥19 U/L) and low ALT group (<19 U/L) based on median of ALT.

Reference

[1]Wang Z, Zhao X, Chen S, Wang Y, Cao L, Liao W, Sun Y, Wang X, Zheng Y, Wu S, Wang L. Associations Between Nonalcoholic Fatty Liver Disease and Cancers in a Large Cohort in China. Clin Gastroenterol Hepatol. 2021 Apr;19(4):788-796.e4. doi: 10.1016/j.cgh.2020.05.009.

[2]Gao X, Fan JG; Study Group of Liver and Metabolism, Chinese Society of Endocrinology. Diagnosis and management of non-alcoholic fatty liver disease and related metabolic disorders: consensus statement from the Study Group of Liver and Metabolism, Chinese Society of Endocrinology. J Diabetes. 2013 Dec;5(4):406-15. doi: 10.1111/1753-0407.12056. Epub 2013 Jun 4.

[3]Xu J, Dai L, Zhang Y, Wang A, Li H, Wang Y, Meng X, Wu S, Wang Y. Severity of Nonalcoholic Fatty Liver Disease and Risk of Future Ischemic Stroke Events. Stroke. 2021 Jan;52(1):103-110. doi: 10.1161/STROKEAHA.120.030433.

[4]Eslam M, Sarin SK, Wong VW, Fan JG, Kawaguchi T, Ahn SH, et al. The Asian Pacific Association for the Study of the Liver clinical practice guidelines for the diagnosis and management of metabolic associated fatty liver disease. Hepatol Int. 2020 Dec;14(6):889-919. doi: 10.1007/s12072-020-10094-2.

[5]Eslam M, Newsome PN, Sarin SK, Anstee QM, Targher G, Romero-Gomez M, et al. A new definition for metabolic dysfunction-associated fatty liver disease: An international expert consensus statement. J Hepatol. 2020 Jul;73(1):202-209. doi: 10.1016/j.jhep.2020.03.039.

[6]Liu T, Zhang Q, Song C et al. C-reactive protein trajectories and the risk of all cancer types: A prospective cohort study. Int J Cancer 2022; 151: 297-307.

[7] Hanley JA. A heuristic approach to the formulas for population attributable fraction. J Epidemiol Community Health. 2001 Jul;55(7):508-14. doi: 10.1136/jech.55.7.508.

© 2023 Liu C et al. *JAMA Network Open.*

eTable 1 Determination of new-onset NAFLD

2006-07	2008-09	2010-11	2012-13	2014-15
0	1			
NA	0	1		
0	NA	1		
0	0	1		
NA	NA	0	1	
NA	0	NA	1	
NA	0	0	1	
0	NA	NA	1	
0	NA	0	1	
0	0	NA	1	
0	0	0	1	
0	0	0	0	1
0	0	0	NA	1
0	0	NA	0	1
0	0	NA	0	1
0	NA	0	0	1
0	NA	0	NA	1
0	NA	NA	0	1
0	NA	NA	NA	1
NA	0	0	0	1
NA	0	0	NA	1
NA	0	NA	0	1
NA	0	NA	NA	1
NA	NA	0	0	1
NA	NA	NA	0	1
NA	NA	0	NA	1

2006-07	2008-09	2010-11	2012-13	2014-15	2016-17
NA	NA	NA	NA	0	1
NA	NA	NA	0	NA	1
NA	NA	NA	0	0	1
NA	NA	0	NA	NA	1
NA	NA	0	NA	0	1
NA	NA	0	0	NA	1
NA	NA	0	0	0	1
NA	0	NA	NA	NA	1
NA	0	NA	NA	0	1
NA	0	NA	0	NA	1
NA	0	NA	0	0	1
NA	0	0	NA	NA	1
NA	0	0	NA	0	1
NA	0	0	0	NA	1
NA	0	0	0	0	1
0	NA	NA	NA	NA	1
0	NA	NA	NA	0	1
0	NA	NA	0	NA	1
0	NA	NA	0	0	1
0	NA	0	NA	NA	1
0	NA	0	NA	0	1
0	NA	0	0	NA	1
0	0	0	0	0	1
0	0	NA	NA	NA	1
0	0	NA	NA	0	1
0	0	NA	0	NA	1
0	0	NA	0	0	1
0	0	0	NA	NA	1
0	0	0	NA	0	1
0	0	0	0	NA	1
0	0	0	0	0	1

NA: The participants did not come to the hospital for medical examination in this cycle.

0: The participants participated in this round of physical examination, but was not diagnosed as NAFLD.

1: The participants participated in this round of physical examination and was diagnosed as NAFLD.

© 2023 Liu C et al. *JAMA Network Open.*

eTable 2 Comparison of baseline characteristics between stayed and excluded participants

Variables	Patients with missing covariate data (Excluded group)	Patients included in the final analysis (Stayed group)	P value (excluding missing data)
N	10240	31848	
Age (year)	51.19(11.67)	51.37(12.43)	0.853
Men (%)	8503(83.2)	26382(82.8)	0.640
Regular Physical activity (%)	1547(15.1)	4874(15.3)	0.630
Current Smoke (%)			0.443 [a]
No	6510(63.6)	25061(78.7)	
Yes	1084(10.6)	6787(21.3)	
Unknown	1928(18.8)	0	
GSD (%)			0.009 [a]
No	8878(86.7)	31122(97.7)	
Yes	251(2.5)	726(2.3)	
Unknown	1111(10.8)	0	
Gallbladder polyps (%)			0.071 [a]
No	8946(87.4)	31300(98.3)	
Yes	183(1.8)	548(1.7)	
Unknown	1111(10.8)	0	
Diabetes (%)			0.075 [a]
No	9150(89.4)	28696(90.1)	
Yes	1074(10.5)	3152(9.9)	
Unknown	16(0.2)	0	
Hypertension (%)	4739(46.3)	14518(45.6)	0.229
BMI			0.111 [a]
<24.0	2352(23.0)	8511(26.7)	
24-27.99	4427(43.2)	16867(53.0)	
≥28	1744(17.0)	6470(20.3)	
Unknown	1717(16.8)	0	
BMI (Kg/m2)	25.81(3.25)	25.78(2.97)	0.233 [a]
WC (cm)	88.35(9.53)	89.22(9.11)	<0.001 [a]
TG (mmol/L)	1.49[1.01,2.51]	1.46[1.02,2.19]	<0.001 [a]
TC (mmol/L)	4.98[4.20,5.72]	5.08[4.49,5.75]	<0.001 [a]
HDL-c (mmol/L)	1.37[1.14,1.66]	1.36[1.14,1.63]	0.377 [a]
hsCRP (mg/L)	1.40[0.60,2.80]	1.40[0.70,2.90]	0.057 [a]
TBil (umol/L)	13.3[10.2,17.2]	13.0[10.2,16.5]	<0.001 [a]
ALT (u/L)	20.0[15.0,27.0]	20.0[15.0,28.0]	<0.001 [a]

[a] Participants with missing values will be excluded when performing group comparisons. For example, for Current Smoke, there were 1928 participants with unknown status. When comparing, these 1928 participants would not be included in the chi-square test.

© 2023 Liu C et al. *JAMA Network Open.*

eTable 3 The number of onset-cases in four groups

		Onset age of NAFLD			
		**<45**	**45-54**	**55-64**	**≥65**
**Esophageal cancer**	Control	2	20	13	8
	NAFLD	5	21	11	10
**Stomach cancer**	Control	7	21	27	22
	NAFLD	5	28	40	24
**Small intestine cancer**	Control	1	4	3	1
	NAFLD	0	2	1	4
**Colorectal cancer**	Control	5	24	56	24
	NAFLD	17	54	59	26
**Liver cancer**	Control	12	23	24	13
	NAFLD	23	27	25	10
**Gallbladder and extrahepatic bile duct cancer**	Control	1	2	6	2
	NAFLD	2	2	6	2
**Pancreatic cancer**	Control	2	7	9	8
	NAFLD	5	6	8	6
**Lung cancer**	Control	21	89	130	88
	NAFLD	26	120	143	75
**Kidney cancer**	Control	2	12	10	8
	NAFLD	2	14	9	1
**Bladder cancer**	Control	2	10	21	17
	NAFLD	3	11	26	17
**Lymphoma and Leukemia**	Control	5	9	10	6
	NAFLD	4	11	7	7
**Breast cancer**	Control	13	38	30	11
	NAFLD	8	37	25	5
**Female reproductive system cancers** [a]	Control	5	21	10	2
	NAFLD	7	13	10	0
**Prostate cancer**	Control	0	1	5	13
	NAFLD	0	3	13	13

[a] Female reproductive system tumors included cervical cancer, endometrial cancer, ovarian cancer, etc.

Other tumors (including head and neck tumors, skin tumors, bone tumors and some special tumors) were not listed in the table.

© 2023 Liu C et al. *JAMA Network Open.*

eTable 4 Subgroup analysis (based on hs-CRP) of the Average Hazard Ratios (95% CI) of cancers among participants with new-onset NAFLD versus controls across age groups.

	hs-CRP<3mg/L (n=49170)		hs-CRP≥3mg/L (n=14526)	
**Age of new-onset NAFLD**	**Average HR (95%CI)**	**P value**	**Average HR (95%CI)**	**P value**
**All cancer types**				
**<45**	1.528(0.991, 2.357)	0.054	1.376(0.789,2.401)	0.261
**45-54**	1.448(1.166,1.800)	0.001	1.837(1.077,3.135)	0.026
**55-64**	1.021(0.772,1.352)	0.882	1.239(1.016,1.512)	0.034
**≥65**	1.026(0.555,1.896)	0.936	0.440(0.286,0.676)	0.001
**P for interaction [a]**	0.058		**0.015**	
**Digestive System Cancers**				
**<45**	1.945(0.870,4.349)	0.105	1.936(0.851,4.406)	0.115
**45-54**	1.786(1.236,2.578)	0.002	2.102(1.157,3.818)	0.014
**55-64**	1.231(0.801,1.599)	0.484	1.002(0.607,1.655)	0.992
**≥65**	1.223(0.798,1.838)	0.335	0.632(0.332,1.203)	0.163
**P for interaction [a]**	0.121		**<0.001**	
**Liver cancer**				
**<45**	2.482(0.874,7.044)	0.087	2.992(0.627,14.308)	0.170
**45-54**	1.316(0.387,4.518)	0.662	2.672(1.268,5.628)	0.010
**55-64**	1.286(0.595,2.781)	0.523	1.010(0.364,3.322)	0.866
**≥65**	1.519(0.460,5.014)	0.492	0.409(0.060,2.687)	0.452
**P for interaction [a]**	0.080		**<0.001**	
**Colorectal cancer**				
**<45**	1.534(0.239,9.842)	0.652	5.438(0.431,48.643)	0.191
**45-54**	2.336(1.158,4.712)	0.018	8.641(1.783,31.888)	0.007
**55-64**	1.164(0.702,1.932)	0.554	0.871(0.387,1.960)	0.739
**≥65**	1.244(0.518,2.986)	0.625	1.242(0.431,3.576)	0.688
**P for interaction [a]**	0.688		**0.024**	
**Lung cancer**				
**<45**	0.731(0.212,2.522)	0.620	2.844(1.202,6.921)	0.017
**45-54**	1.721(0.893,3.315)	0.104	1.873(1.156,3.035)	0.011
**55-64**	1.152(0.810,1.636)	0.430	0.986(0.596,1.631)	0.956
**≥65**	0.605(0.282,1.300)	0.198	0.569(0.283,1.148)	0.116
**P for interaction [a]**	0.151		**0.017**	

The model was adjusted for age (continuous), sex (categorical), BMI (continuous), waist circumference (continuous), TC (continuous), TG (continuous), Tbil (continuous), hsCRP (continuous), ALT (continuous), smoking status (categorical), physical activity (categorical), Hypertension (categorical), Diabetes (categorical), Gallbladder polyps (categorical), GSD (categorical).

[a] The interaction between NAFLD and its age at diagnosis.

© 2023 Liu C et al. *JAMA Network Open.*

eTable 5 Subgroup analysis (based on ALT) of the Average Hazard Ratios (95% CI) of cancers among participants with new-onset NAFLD versus controls across age groups.

	ALT<19 U/L (n=31838)		ALT≥19 U/L (n=31858)	
**Age of new-onset NAFLD**	**Average HR (95%CI)**	**P value**	**Average HR (95%CI)**	**P value**
**All cancer types**				
**<45**	1.088(0.685,1.73)	0.720	1.912(1.174,3.113)	0.009
**45-54**	1.557(1.228,1.1975)	<0.001	1.375(1.038,2.176)	0.029
**55-64**	1.144(0.886,1.477)	0.304	1.259(0.998,1.588)	0.052
**≥65**	1.199(0.907,1.585)	0.203	0.558(0.345,0.905)	0.018
**P for interaction [a]**	0.104		**0.005**	
**Digestive System Cancers**				
**<45**	1.519(0.692,3.513)	0.284	2.792(1.816,4.294)	<0.001
**45-54**	2.302(1.110,4.774)	0.025	1.559(0.693,3.507)	0.283
**55-64**	0.923(0.634,1.343)	0.675	1.322(0.874,1.997)	0.186
**≥65**	1.221(0.760,1.960)	0.409	0.765(0.476,1.232)	0.271
**P for interaction [a]**	**0.028**		**0.009**	
**Liver cancer**				
**<45**	8.664(1.700,44.163)	0.009	1.322(0.406,4.301)	0.642
**45-54**	6.471(2.020,20.724)	0.001	1.149(0.530,2.492)	0.724
**55-64**	1.674(0.552,5.049)	0.360	1.086(0.515,2.289)	0.829
**≥65**	0.971(0.242,3.904)	0.967	0.740(0.340,1.607)	0.446
**P for interaction [a]**	**<0.001**		**<0.001**	
**Colorectal cancer**				
**<45**	0.676(0.19,2.404)	0.545	3.021(3.630,25.144)	0.002
**45-54**	4.701(2.153,10.266)	<0.001	2.092(0.914,4.790)	0.081
**55-64**	0.900(0.524,1.548)	0.704	1.332(0.721,2.492)	0.369
**≥65**	1.455(0.685,3.091)	0.330	0.958(0.277,3.308)	0.946
**P for interaction [a]**	0.989		**<0.001**	
**Lung cancer**				
**<45**	1.412(0.445,4.482)	0.558	2.488(0.935,6.622)	0.068
**45-54**	1.350(0.789,2.310)	0.273	2.112(1.081,4.128)	0.028
**55-64**	0.968(0.654,1.431)	0.869	1.327(0.858,2.052)	0.204
**≥65**	0.939(0.598,1.476)	0.786	0.478(0.197,1.159)	0.102
**P for interaction [a]**	0.123		**0.016**	

The model was adjusted for age (continuous), sex (categorical), BMI (continuous), waist circumference (continuous), TC (continuous), TG (continuous), Tbil (continuous), hsCRP (continuous), ALT (continuous), smoking status (categorical), physical activity (categorical), Hypertension (categorical), Diabetes (categorical), Gallbladder polyps (categorical), GSD (categorical).

[a] The interaction between NAFLD and its age at diagnosis.

© 2023 Liu C et al. *JAMA Network Open.*

eTable 6 The association of new-onset NAFLD with the risk of cancers in competing risk analysis

Age of new-onset NAFLD	SD	P value	CS	P value
	HR (95%CI)		HR (95%CI)	
**All cancer types**				
**<45**	1.356(1.306,1.776)	0.026	1.359(1.034,1.787)	0.028
**45-54**	1.069(0.875,1.306)	0.516	1.231(1.068,1.418)	0.004
**55-64**	1.121(0.981,1.280)	0.092	1.122(0.982,1.282)	0.091
**≥65**	0.933(0.784,1.110)	0.438	0.933(0.785,1.111)	0.433
**Digestive System Cancers**				
**<45**	1.813(1.159,2.835)	0.009	1.815(1.152,2.859)	0.010
**45-54**	1.436(1.105,1.865)	0.007	1.440(1.110,1.869)	0.006
**55-64**	1.179(0.931,1.492)	0.171	1.179(0.935,1.486)	0.164
**≥65**	1.041(0.760,1.426)	0.802	1.046(0.761,1.438)	0.783
**Liver cancer**				
**<45**	1.632(0.810,3.286)	0.171	1.635(0.805,3.320)	0.174
**45-54**	1.172(0.664,2.066)	0.584	1.178(0.675,2.056)	0.565
**55-64**	1.086(0.615,1.919)	0.775	1.089(0.614,1.930)	0.771
**≥65**	0.723(0.304,1.722)	0.464	0.724(0.314,1.668)	0.447
**Colorectal cancer**				
**<45**	3.333(1.210,9.184)	0.020	3.333(1.211,9.176)	0.020
**45-54**	2.242(1.375,3.656)	0.001	2.250(1.379,3.671)	0.001
**55-64**	1.241(0.864,1.783)	0.242	1.243(0.868,1.779)	0.235
**≥65**	1.074(0.613,1.879)	0.804	1.087(0.614,1.923)	0.775
**Lung cancer**				
**<45**	1.538(0.834,2.836)	0.168	1.546(0.846,2.823)	0.156
**45-54**	1.335(1.023,1.128)	0.043	1.342(1.010,1.784)	0.040
**55-64**	1.127(0.884,1.437)	0.333	1.128(0.885,1.436)	0.330
**≥65**	0.932(0.684,1.271)	0.657	0.936(0.685,1.280)	0.679

The model was adjusted for age (continuous), sex (categorical), BMI (continuous), waist circumference (continuous), TC (continuous), TG (continuous), Tbil (continuous), hsCRP (continuous), ALT (continuous), smoking status (categorical), physical activity (categorical), Hypertension (categorical), Diabetes (categorical), Gallbladder polyps (categorical), GSD (categorical).

© 2023 Liu C et al. *JAMA Network Open.*

eTable 7 The Average Hazard Ratios (95% CI) of overall cancers among participants with new-onset NAFLD versus controls across age groups.（Excluding participants with follow-up less than 1 year, n=63176）

Age of new-onset NAFLD	Average HR (95%CI)	P value
**All cancer types**		
**<45**	1.570(1.191,2.070)	<0.001
**45-54**	1.532(1.084,2.165)	0.016
**55-64**	1.161(0.979,1.376)	0.086
**≥65**	0.782(0.445,1.375)	0.393
**P for interaction [a]**	**0.002**	
**Digestive System Cancers**		
**<45**	1.970(1.422,2.730)	<0.001
**45-54**	1.942(1.422,2.270)	0.030
**55-64**	1.084(0.809,1.453)	0.590
**≥65**	0.720(0.498,1.042)	0.082
**P for interaction [a]**	**0.002**	
**Liver cancer**		
**<45**	2.896(1.252,6.698)	0.013
**45-54**	2.226(1.174,4.220)	0.014
**55-64**	1.343(0.706,2.554)	0.368
**≥65**	0.506(0.221,1.158)	0.107
**P for interaction [a]**	**<0.001**	
**Colorectal cancer**		
**<45**	2.054(0.475,8.871)	0.335
**45-54**	3.042(1.636,5.658)	<0.001
**55-64**	1.054(0.684,1.625)	0.811
**≥65**	1.075(0.517,2.236)	0.847
**P for interaction [a]**	0.242	
**Lung cancer**		
**<45**	2.270(1.085,4.747)	0.029
**45-54**	1.968(1.084,3.572)	0.026
**55-64**	1.121(0.839,1.520)	0.439
**≥65**	0.653(0.353,1.207)	0.173
**P for interaction [a]**	**0.006**	

The model was adjusted for age (continuous), sex (categorical), BMI (continuous), waist circumference (continuous), TC (continuous), TG (continuous), Tbil (continuous), hsCRP (continuous), ALT (continuous), smoking status (categorical), physical activity (categorical), Hypertension (categorical), Diabetes (categorical), Gallbladder polyps (categorical), GSD (categorical).

[a] The interaction between NAFLD and its age at diagnosis.

© 2023 Liu C et al. *JAMA Network Open.*

eTable 8 The Average Hazard Ratios (95% CI) of overall cancers among participants with new-onset NAFLD versus controls across age groups.（Excluding participants who take regular physical activity , n=56651, excluding participants who regularly take lipid-lowering drugs, n=52536, and exclude participants who with a late diagnosis of NAFLD, n=62595）

Age of new-onset NAFLD	Excluding participants who take regular physical activity [a]	P value	Excluding participants who regularly take lipid-lowering drugs [a]	P value	Exclude participants who with a late diagnosis of NAFLD [b]	P value	Additional adjusting for stages of steatosis and lifestyle factors [c]	P value
	Average HR (95%CI)		Average HR (95%CI)		Average HR (95%CI)		Average HR (95%CI)	
All cancer types								
<45	1.382(0.974,1.963)	0.069	1.512(1.070,2.102)	0.019	1.453 (1.035,2.040)	0.031	1.515 (1.081,2.123)	0.016
45-54	1.344(1.105,1.635)	0.003	1.552(1.177,2.047)	0.002	1.564 (1.180,2.072)	0.002	1.506 (1.144,1.981)	0.004
55-64	1.241(1.036,1.486)	0.018	1.189(1.006,1.406)	0.042	1.189 (1.007,1.404)	0.042	1.168 (0.990,1.378)	0.066
≥65	1.06(0.838,1.340)	0.626	0.734(0.428,1.260)	0.263	0.724 (0.421,1.245)	0.243	0.763 (0.456,1.278)	0.304
Digestive System Cancers								
<45	1.781(0.986,3.219)	0.055	2.023(1.135,3.607)	0.017	1.907 (1.079,3.372)	0.026	2.077 (1.169,3.691)	0.013
45-54	1.874(1.334,2.631)	<0.001	2.008(1.413,2.855)	<0.001	1.954 (1.419,2.693)	<0.001	1.837 (1.348,2.502)	<0.001
55-64	1.216(0.898,1.645)	0.207	1.204(0.871,1.664)	0.262	1.080 (0.812,1.437)	0.595	1.075 (0.804,1.437)	0.625
≥65	1.520(0.999,2.312)	0.050	0.682(0.445,1.045)	0.078	0.652 (0.455,1.037)	0.075	0.719 (0.503,1.028)	0.071
Liver cancer								
<45	2.327(1.022,5.297)	0.044	3.087(1.306,7.301)	0.010	2.675 (1.216,5.882)	0.014	2.831 (1.302,6.156)	0.009
45-54	2.232(1.054,4.722)	0.036	2.005(1.047,3.840)	0.036	2.475 (1.294,4.731)	0.006	2.132 (1.128,4.029)	0.020
55-64	1.474(0.753,2.881)	0.257	1.529(0.744,3.141)	0.248	1.249 (0.682,2.289)	0.471	1.318 (0.712,2.441)	0.379
≥65	2.061(0.725,5.858)	0.174	0.427(0.177,1.036)	0.059	0.510 (0.251,1.042)	0.065	0.645 (0.281,1.483)	0.302
Colorectal cancer								
<45	2.002(0.483,8.301)	0.339	1.991(0.482,8.227)	0.341	1.818 (0.576,6.934)	0.382	1.974 (0.491,7.943)	0.338
45-54	3.080(1.597,5.893)	0.001	4.307(2.297,8.076)	<0.001	2.933 (1.591,5.406)	<0.001	2.938 (1.601,5.392)	0.001
55-64	1.204(0.770,1.772)	0.416	1.166(0.721,1.885)	0.531	1.012 (0.659,1.554)	0.956	1.026 (0.663,1.588)	0.907

© 2023 Liu C et al. *JAMA Network Open.*

**≥65**	1.283(0.587,2.806)	0.532	1.455(0.715,2.963)	0.301	1.137 (0.577,2.240)	0.711	1.139 (0.592,2.192)	0.696
**Lung cancer**								
**<45**	2.928(1.410,6.081)	0.004	3.397(1.594,7.241)	0.002	2.953 (1.448,6.019)	0.003	2.913 (1.431,5.932)	0.003
**45-54**	1.267(0.808,1.986)	0.302	2.087(1.067,4.082)	0.032	2.078 (1.050,4.113)	0.036	2.021 (1.038,3.936)	0.038
**55-64**	1.129(0.828,1.539)	0.443	1.158(0.831,1.616)	0.386	1.154 (0.867,1.537)	0.325	1.126 (0.852,1.489)	0.405
**≥65**	1.061(0.671,1.676)	0.801	0.524(0.257,1.068)	0.075	0.618 (0.308,1.242)	0.177	0.612 (0.324,1.160)	0.133

[a] The model was adjusted for age (continuous), sex (categorical), BMI (continuous), waist circumference (continuous), TC (continuous), TG (continuous), Tbil (continuous), hsCRP (continuous), ALT (continuous), smoking status (categorical), physical activity (categorical), Hypertension (categorical), Diabetes (categorical), Gallbladder polyps (categorical), GSD (categorical).

[b] A late diagnosis of NAFLD is considered to be severe NAFLD or cirrhosis.

[c] The model was adjusted for age (continuous), sex (categorical), BMI (continuous), waist circumference (continuous), TC (continuous), TG (continuous), Tbil (continuous), hsCRP (continuous), ALT (continuous), smoking status (categorical), physical activity (categorical), Hypertension (categorical), Diabetes (categorical), Gallbladder polyps (categorical), GSD (categorical), stages of steatosis (categorical), sedentary (categorical), high-fat diet (categorical), salt intake (categorical).

© 2023 Liu C et al. *JAMA Network Open.*

eTable 9 The Average Hazard Ratios (95% CI) of overall cancers among participants with new-onset NAFLD versus controls across age groups after PSM（n=53778）

Age of new-onset NAFLD	Average HR (95%CI)	P value
**All cancer types**		
**<45**	1.514(1.162,1.971)	0.002
**45-54**	1.511(1.081,2.112)	0.015
**55-64**	1.175(0.977,1.385)	0.054
**≥65**	0.754(0.444,1.280)	0.296
**P for interaction [a]**	**0.004**	
**Digestive System Cancers**		
**<45**	2.086(1.136,3.831)	
**45-54**	1.878(1.377,2.561)	<0.001
**55-64**	1.082(0.816,1.434)	0.581
**≥65**	0.707(0.620,1.003)	0.051
**P for interaction [a]**	**0.019**	
**Liver cancer**		
**<45**	2.355(1.037,5.347)	0.040
**45-54**	2.203(1.888,4.085)	0.012
**55-64**	1.245(0.684,2.268)	0.472
**≥65**	0.276(NA)	
**P for interaction [a]**	**0.001**	
**Colorectal cancer**		
**<45**	2.033(0.477,8.667)	0.337
**45-54**	2.988(1.630,5.477)	<0.001
**55-64**	1.045(0.686,1.594)	0.836
**≥65**	1.116(0.571,2.187)	0.748
**P for interaction [a]**	0.240	
**Lung cancer**		
**<45**	2.154(1.052,4.409)	0.035
**45-54**	1.980(1.103,3.553)	0.022
**55-64**	1.132(0.851,1.505)	0.396
**≥65**	0.642(0.344,1.199)	0.164
**P for interaction [a]**	**0.006**	

The model was adjusted for age (continuous), sex (categorical), BMI (continuous), waist circumference (continuous), TC (continuous), TG (continuous), Tbil (continuous), hsCRP (continuous), ALT (continuous), smoking status (categorical), physical activity (categorical), Hypertension (categorical), Diabetes (categorical), Gallbladder polyps (categorical), GSD (categorical).

[a] The interaction between NAFLD and its age at diagnosis.

© 2023 Liu C et al. *JAMA Network Open.*

eTable 10 The Average Hazard Ratios (95% CI) of overall cancers among participants with new-onset MAFLD versus controls across age groups.（n=61712）

Age of new-onset MAFLD	Average HR (95%CI)	P value
**All cancer types**		
**<45**	1.493(1.063,2.100)	0.021
**45-54**	1.480(1.122,1.952)	0.006
**55-64**	1.165(0.987,1.377)	0.071
**≥65**	0.640(0.369,1.112)	0.113
**P for interaction [a]**	**<0.001**	
**Digestive System Cancers**		
**<45**	1.854(1.333,2.576)	<0.001
**45-54**	1.851(1.040,3.297)	0.036
**55-64**	1.020(0.866,1.356)	0.792
**≥65**	0.730(0.514,1.038)	0.078
**P for interaction [a]**	**0.002**	
**Liver cancer**		
**<45**	2.362(1.132,4.928)	0.022
**45-54**	2.131(1.138,3.991)	0.018
**55-64**	1.109(0.604,2.040)	0.738
**≥65**	0.569(0.278,1.167)	0.124
**P for interaction [a]**	**<0.001**	
**Colorectal cancer**		
**<45**	2.033(0.472,8.754)	0.341
**45-54**	3.908(1.681,5.931)	0.009
**55-64**	0.971(0.631,1.494)	0.894
**≥65**	1.178(0.589,2.354)	0.642
**P for interaction [a]**	0.114	
**Lung cancer**		
**<45**	2.015(1.042,4.663)	0.039
**45-54**	1.894(1.000,3.590)	0.050
**55-64**	1.139(0.849,1.530)	0.385
**≥65**	0.617(0.351,1.087)	0.095
**P for interaction [a]**	**0.009**	

The model was adjusted for age (continuous), sex (categorical), BMI (continuous), waist circumference (continuous), TC (continuous), TG (continuous), Tbil (continuous), hsCRP (continuous), ALT (continuous), smoking status (categorical), physical activity (categorical), Hypertension (categorical), Diabetes (categorical), Gallbladder polyps (categorical), GSD (categorical).

[a] The interaction between NAFLD and its age at diagnosis.

© 2023 Liu C et al. *JAMA Network Open.*

eFigure 1 The Average Hazard Ratios (95% CI) of specific cancers among participants with new-onset NAFLD versus controls across age groups.

Onset Age year of NAFLD	Average Hazard Ratio (95%CI)	P-value
**Liver Cancer**	**(P for interaction<0.001)**	
<45	2.655(1.155,6.106)	0.022
45-54	2.159(1.148,4.061)	0.017
55-64	1.273(0.695,2.330)	0.434
≥65	0.567(0.278,1.157)	0.119
**Colorectal Cancer**	**(P for interaction<0.001)**	
<45	2.054(0.675,8.871)	0.335
45-54	2.894(1.428,5.505)	<0.001
55-64	1.045(0.686,1.592)	0.836
≥65	0.949(0.547,2.161)	0.794
**Esophageal Cancer**	**(P for interaction=0.757)**	
<45	2.176(0.314,15.082)	0.431
45-54	1.076(0.542,2.134)	0.834
55-64	0.688(0.310,1.525)	0.357
≥65	1.035(0.542,3.387)	0.516
**Gastric Cancer**	**(P for interaction=0.691)**	
<45	0.766(0.218,2.309)	0.569
45-54	1.702(0.960,3.016)	0.068
55-64	1.804(0.973,3.342)	0.061
≥65	1.399(0.688,2.846)	0.354
**Small intestine cancer**	**(P for interaction=0.215)**	
<45	NA(NA,NA)	
45-54	0.622(0.101,3.821)	0.608
55-64	0.257(0.017,3.806)	0.323
≥65	NA(NA,NA)	
**Gallbladder and extrahepatic bile duct cancer**	**(P for interaction=0.224)**	
<45	NA(NA,NA)	
45-54	3.167(0.401,25.012)	0.274
55-64	1.396(0.467,4.169)	0.550
≥65	1.159(0.127,10.57)	0.896
**Pancreatic cancer**	**(P for interaction=0.347)**	
<45	2.662(0.554,13.274)	0.232
45-54	0.734(0.218,2.113)	0.619
55-64	0.548(0.142,2.113)	0.382
≥65	1.047(0.322,3.416)	0.939

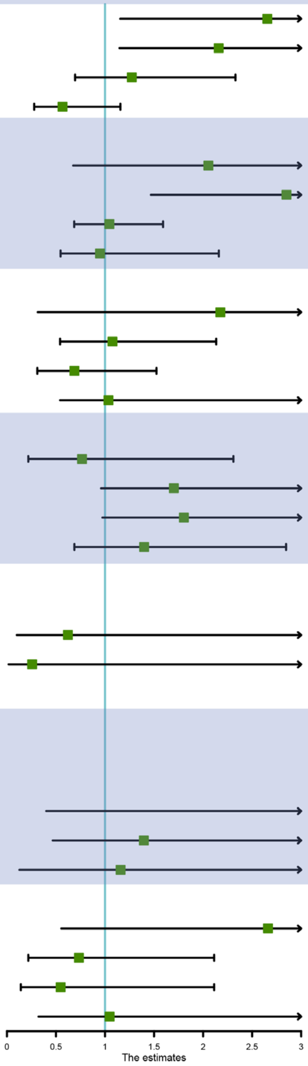

© 2023 Liu C et al. *JAMA Network Open.*

Onset Age year of NAFLD	Average Hazard Ratio (95%CI)	P-value
**Lung Cancer**	**(P for interaction=0.006)**	
<45	2.137(1.046,4.364)	0.037
45-54	1.972(1.010,3.537)	0.023
55-64	1.131(0.851,1.304)	0.396
≥65	0.632(0.344,1.162)	0.140
**Breast Cancer**	**(P for interaction=0.497)**	
<45	0.669(0.282,1.583)	0.360
45-54	1.026(0.585,1.798)	0.929
55-64	1.088(0.596,1.986)	0.784
≥65	0.810(0.222,2.957)	0.750
**Female reproductive system cancer**	**(P for interaction=0.742)**	
<45	1.181(0.318,4.387)	0.804
45-54	0.689(0.322,1.471)	0.335
55-64	1.172(0.374,3.668)	0.785
≥65	NA(NA,NA)	
**Prostatic Cancer**	**(P for interaction=0.221)**	
<45	NA(NA,NA)	
45-54	2.030(0.189,21.807)	0.515
55-64	4.377(1.335,14.351)	0.015
≥65	0.684(0.235,1.991)	0.564
**Urinary system cancer**	**(P for interaction=0.181)**	
<45	1.484(0.379,5.822)	0.570
45-54	1.113(0.557,2.224)	0.761
55-64	0.905(0.485,1.688)	0.753
≥65	0.563(0.267,1.175)	0.125
**Lymphoma and leukemia**	**(P for interaction=0.330)**	
<45	1.720(0.450,6.570)	0.428
45-54	1.571(0.609,4.054)	0.350
55-64	0.574(0.211,4.560)	0.277
≥65	1.226(0.387,3.880)	0.729
**Other cancer types**	**(P for interaction=0.340)**	
<45	0.790(0.388,1.608)	0.515
45-54	1.098(0.760,1.586)	0.619
55-64	1.535(0.988,2.384)	0.057
≥65	1.864(1.138,3.052)	0.013

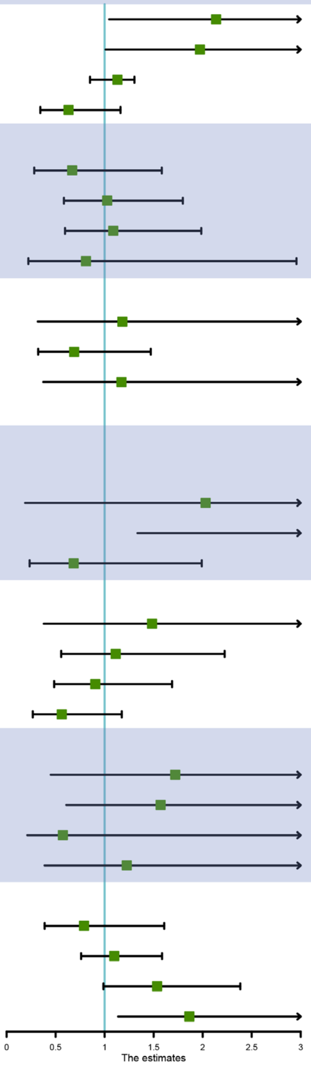

© 2023 Liu C et al. *JAMA Network Open.*

eFigure 2 Populations Attributable Fractions (PAFs) of cancer associated with new-onset NAFLD in different ages

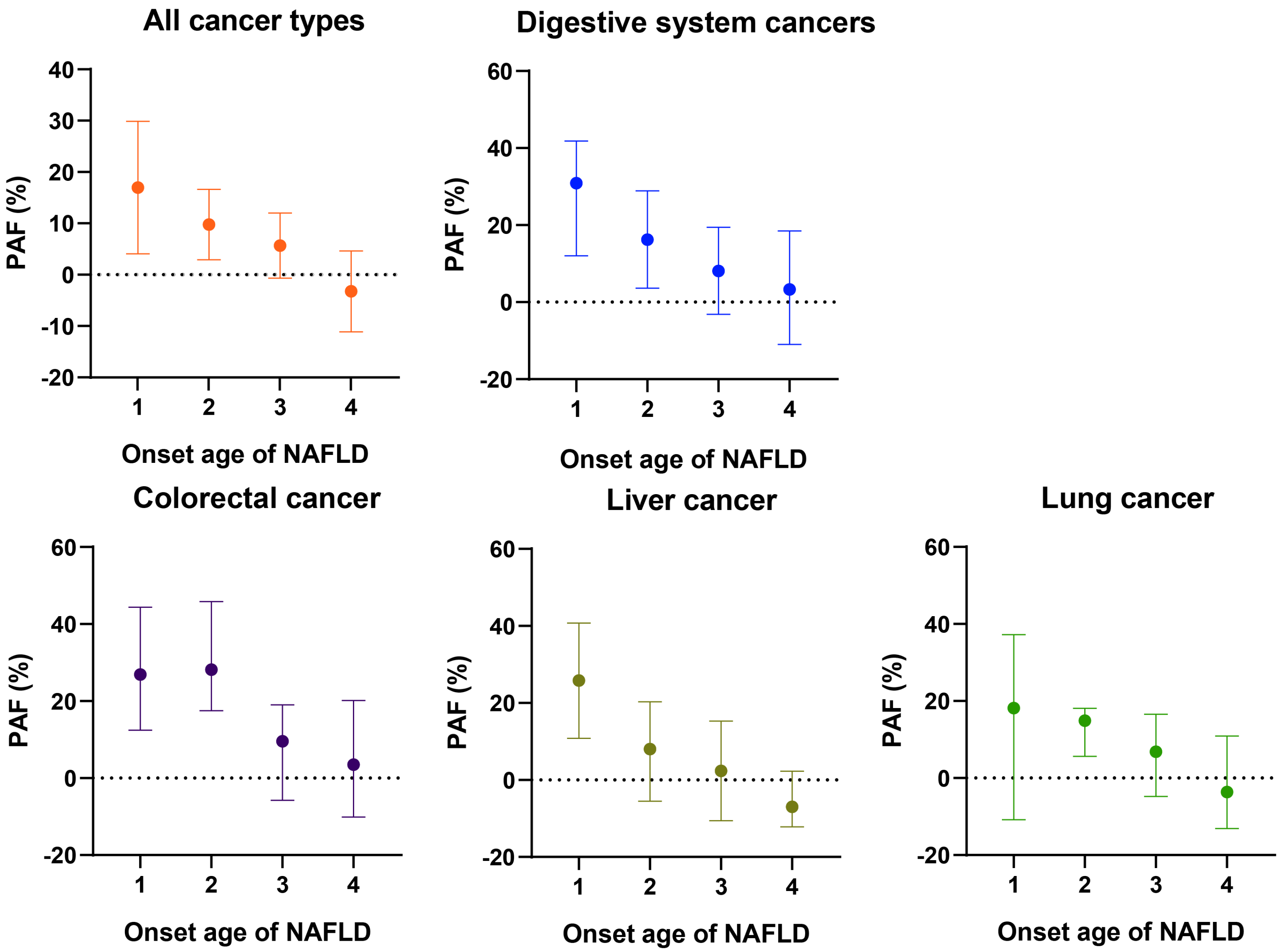

© 2023 Liu C et al. *JAMA Network Open.*
