# Supplementary material for: New-Onset Age of Nonalcoholic Fatty Liver Disease and Cancer Risk
Source: JAMA Netw Open. 2023 Sep 25;6(9):e2335511. doi: 10.1001/jamanetworkopen.2023.35511 (PMC10520743; doi:10.1001/jamanetworkopen.2023.35511)
Supplement: Supplement 2. — Data Sharing Statement [file jamanetwopen-e2335511-s002.pdf]

Data Sharing Statement

Liu C. New-Onset Age of Nonalcoholic Fatty Liver Disease and Cancer Risk. *JAMA Netw Open*. Published online September 25, 2023. doi:10.1001/jamanetworkopen.2023.35511

Data
Data available: No
